# Drawings as tools to (re)imagine space in interdisciplinary global health research

**DOI:** 10.3389/fpubh.2022.985430

**Published:** 2022-12-05

**Authors:** Stefanie Dens, Claudia Nieto-Sanchez, Mario De Los Santos, Thomas Hawer, Asgedom Haile, Karla Solari, Jesus Cisneros, Victor Vega, Kalkidan Solomon, Adamu Addissie, Delenasaw Yewhalaw, Larissa Otero, Koen Peeters Grietens, Kristien Verdonck, Maarten Van Acker

**Affiliations:** ^1^Research Group for Urban Development, University of Antwerp, Antwerp, Belgium; ^2^Department of Public Health, Institute of Tropical Medicine, Antwerp, Belgium; ^3^Faculty of Architecture, Pontificia Universidad Católica del Perú, Lima, Peru; ^4^Business Unit Coast, Rivers and Cities, Witteveen+Bos, Antwerp, Belgium; ^5^School of Public Health, Addis Ababa University, Addis Ababa, Ethiopia; ^6^Faculty of Social Sciences, Universidad Nacional Mayor de San Marcos, Lima, Peru; ^7^Instituto de Medicina Tropical Alexander von Humboldt, Universidad Peruana Cayetano Heredia, Lima, Peru; ^8^Tropical and Infectious Diseases Research Center, Jimma University, Jimma, Ethiopia; ^9^School of Medical Laboratory Sciences, Faculty of Health Sciences, Jimma University, Jimma, Ethiopia; ^10^School of Tropical Medicine and Global Health, Nagasaki University, Nagasaki, Japan

**Keywords:** urbanism, epidemiology, ethnography, drawings, airborne diseases, vector-borne diseases, interdisciplinary research, public health

## Abstract

Understanding the role of space in infectious diseases' dynamics in urban contexts is key to developing effective mitigation strategies. Urbanism, a discipline that both studies and acts upon the city, commonly uses drawings to analyze spatial patterns and their variables. This paper revisits drawings as analytical and integrative tools for interdisciplinary research. We introduce the use of drawings in two interdisciplinary projects conducted in the field of global public health: first, a study about the heterogeneous burden of tuberculosis and COVID-19 in Lima, Peru, and second, a study about urban malaria in Jimma, Ethiopia. In both cases, drawings such as maps, plans, and sections were used to analyze spatial factors present in the urban context at different scales: from the scale of the territory, the city, and the district, to the neighborhood and the household. We discuss the methodological approaches taken in both cases, considering the nature of the diseases being investigated as well as the natural and social context in which the studies took place. We contend that the use of drawings helps to reimagine space in public health research by adding a multidimensional perspective to spatial variables and contexts. The processes and products of drawing can help to (a) identify systemic relations within the spatial context, (b) facilitate integration of quantitative and qualitative data, and (c) guide the formulation of policy recommendations, informing public and urban health planning.

## Introduction

Historically, the discipline of urbanism, simultaneously studying and acting upon the city, has actively contributed to the design of preventive and control measures for infectious diseases in urban contexts (1). In 400 BC, for example, the physician and political advisor, Oribasius, advised that city streets could be designed parallel to each other to facilitate wind circulation and clean the street network of smoke, polluted air, and infectious substances (2). In the nineteenth century, the bubonic plague was defeated by urban health infrastructures such as sewage systems and waste management (3, 4). In the early twentieth century, following high tuberculosis rates in Europe, the modern architecture movement promoted designs that allowed generous light and sun into residences (3). In developing these strategies in urban contexts, the tools of urbanism and, more specifically, drawings, have been instrumental to public health.

**Figure 1:**
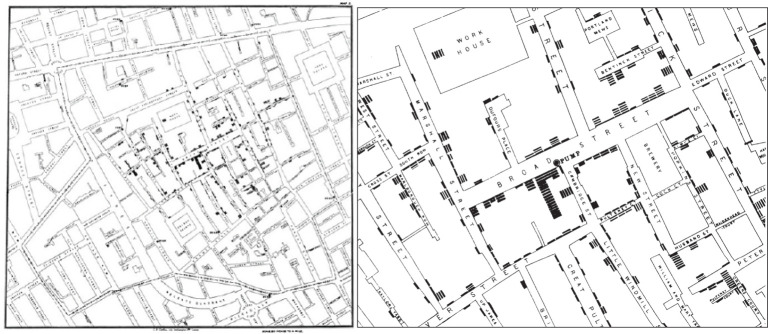
The famous Cholera Map of Broad Street with deaths mapped as black rectangles, Soho, London, 1854 (5).

Maps, in particular, have played a vital role in unraveling the mysteries of how diseases spread (4). The 1854 London Cholera map (Figure 1) is a classic example. At the time, London faced a series of cholera outbreaks and there were intense debates about how this problem should be tackled. During an outbreak in September 1854 in the Soho District, 500 people died within a period of 10 days, after which the English physician John Snow decided to map each occurrence of illness and death. This mapping exercise localized a concentration of 83 cases within a distance of 250 yards around a water pump on Broad Street supplied by the Southwark and Vauxhall Company (6). After analyzing cholera mortality as a function of the supply companies, the pump was identified as an outlet of a polluted well that was tapped into by the water supplier. The strength of this methodology was that the drawing, in this case the map, was used to visualize and locate the collected data that supported the formulation of the hypothesis of cholera being waterborne. This historical example illustrates how urban and medical thinking came together, and how the map created a common ground for decision-making in public health.

In the twenty first century, the role of explaining spatial complexity in disease transmission has been taken over by methodologies developed in the field of spatial epidemiology. Technological developments in geographical information systems (GIS) have accelerated the capture and analysis of important amounts of individual and aggregate data points that can be linked to specific geographical areas (7). These data are often used to visualize the distribution and clustering of disease. However, GIS generated data has limited capacity to integrate contextual factors that can dramatically change the inferences and predictions made through these methods (8). This limitation becomes even more important for highly heterogeneous spatial contexts, such as the ones associated to current urbanization processes.

With the emergence of the COVID-19 pandemic, for which a pharmaceutical solution was not immediately available, space-based first-line containment strategies such as closing down public spaces, stay-home orders, quarantine and distancing measures reinvigorated the debate around spatial complexity. Drawings also made their reentrance into the public health domain as instruments of visualization and communication of prevention measures and regulations, including social-distancing grids, queue lanes, temporary bicycle lanes, COVID-19 safe pedestrian areas and pop-up outdoor dining spaces. Drawings supported politicians and medical staff in the explanation and communication of how airborne disease transmission worked and could be prevented. The urban context, and its public and private realms, were perceived both as the problem [an *epidemic space* (9)], as well as the solution (the immediate mitigation resource), and drawings seemed well placed to reinterpret this tension.

In this paper, we present drawings from the field of urbanism as methodological tools to capture and analyze the complexity of urban realities in global health research. We discuss our experience with different types of drawings in studies about the heterogenous distribution of tuberculosis and COVID-19 across districts in Lima, Peru, and socio-spatial variations potentially involved in malaria persistence in Jimma, Ethiopia. The methodological approach hereby presented is rooted in a socio-ecological perspective applied through the collection and integration of spatial, epidemiological, entomological, and social data at multiple levels (XL – the city, L – the district, M – the neighborhood and the urban tissue, S- the home, and XS – the individual). We contend that this layered examination of spatial realities can more accurately describe epidemic risk in urban contexts by contextualizing general epidemiological trends (usually recorded around large geographical areas, i.e. the city or the district) to more micro levels of analysis in which social practices, landscape, building cultures, and ecology interact to alter spatial realities.

## Methods

In this paper, we show how drawings, as established tools of design research in the domain of urbanism (10), can contribute to interdisciplinary global health research. The domain of urbanism is defined as “an interdisciplinary approach that engages in real-world sociocultural, ecological and technological issues affecting urban landscapes, from the perspective of spatial planning and design” (11). Specifically, urbanism combines the disciplines of spatial planning, urban design, and landscape architecture (11). As such, the drawings discussed in this paper are part of those disciplines, and “drawing”, both a noun and a verb, is considered as a process and a product.

Drawings are used to analyze existing urban contexts (precedent analysis) (11) or to synthesize them by superimposing layers to spatialize and localize information (12). They often cross different scales (13), aiming to find systemic relations by zooming in and out of the urban context, and, in that process, aim to localize opportunities for strategic urban design projects (14). Maps, more specifically, facilitate a “spatial understanding of objects, concepts, conditions, processes or events in the human/natural world” (15). As such, drawing, and mapping in particular, is an activity of constructing and communicating spatial knowledge (11) that constantly transitions between “reading” and “writing” the territory (16). As a process, drawing oscillates between simplification and compilation (14) in the characterization of urban contexts. As products, drawings resulting from that process are not one-to-one copies of the existing situation (11, 16); they are illustrations, compilations and interpretations of spatial factors of interest. Drawing, hence, is an “analytical instrument” (14), “a tool for thinking” (11) and an “instrument for discussion” (14) and integration.

### Sites and approach

#### Case 1: Airborne diseases in Lima, Peru

Peru was one of the countries more severely impacted by the first waves of COVID-19 in Latin America and it is also one of the countries with highest burden of tuberculosis (TB) in the region of the Americas. The capital, Lima, carries a disproportionately high share of the burden of these airborne infectious diseases. Lima houses approximately 11 million people—about one third of the Peruvian population—and is estimated to carry 59% of the country's TB burden (17) and 57% of the COVID-19 burden (18). Lima and Callao (further referred to as Lima) are administratively divided into 50 districts, some of which have a markedly higher TB and COVID-19 burden than others. The aim of this study was to investigate underlying factors involved in the geographical overlap of disease burden in different districts of the city.

This study followed a convergent parallel mixed methods design [(QUAL) + (QUAN)] in which both strands constantly informed each other. Following an ecological design that used the administrative districts as unit of analysis, the quantitative strand used publicly available data and health program data to map the geographical distribution of TB and COVID-19 disease burden and to explore correlations with explanatory variables potentially involved in this distribution. The two outcome variables (TB and COVID-19 disease burden) were expressed as directly standardized rates, i.e. the numbers of cases per 100,000 residents after adjustment for the districts' population structure in terms of age and gender. For COVID-19, the calculations were based on all the deaths attributed to COVID-19 in the national death registry during the first wave (March – November 2020) and the second wave (December 2020 – September 2021) (19). For TB, the calculations were based on the notified cases of active TB in 2018 and 2019 (before the pandemic), without counting cases notified in prisons (20). The explanatory variables were a set of 24 sociodemographic and urban factors. In an early exploratory phase, we used Spearman's rho to assess simple bivariate correlations, i.e., to estimate the strength of association between district characteristics and disease burden. The preliminary findings served as a starting point for urban evaluations (presented in the present manuscript) and for a formal statistical analysis using a Bayesian approach, which allows to jointly assess patterns of TB and COVID-19 while simultaneously accounting for space and other covariates (presented elsewhere).

The qualitative strand generated in-depth (primary) ethnographic data on disease exposure. Taking the quantitative data of these 50 districts as a reference, we theoretically selected six districts (La Victoria, Cercado de Lima, Villa Maria del Triunfo, Callao, Cieneguilla, and San Borja) for in-depth qualitative research. These districts showed variation in the burden of disease, but also different geographical, social and spatial characteristics. For example, while La Victoria and Cercado the Lima are located close to the administrative and commercial center of Lima, Cieneguilla and Callao expand over coastal and mountainous areas that extend the confines of the city. La Victoria, Villa Maria del Triunfo, and Cieneguilla are relatively recent examples of human settlements created and organized to respond the demands of accelerated urbanization processes, while Cercado de Lima and San Borja are contemporaneous expressions of architectural forms that date back to the very foundation of the city. Environmental degradation derived from commercial activities at the port in Callao contrast with the touristic nature of areas next to the sea in Cieneguilla. Uses of space, access to health care, income-generation activities and mobility routines were described for each district and linked to domestic routines and living conditions at the household level. These initial findings were further explored through a desk review and primary data collection from an urbanism perspective. The results presented here integrate epidemiological, social, and urban data.

#### Case 2: Vector-borne diseases in Jimma, Ethiopia

In the city of Jimma in Ethiopia, malaria transmission persists despite extended adoption of preventive measures. Spatial variables such as proximity to stagnant water and modified environments resulting from urban interventions have been previously associated with malaria in this region (21). Since urbanization rates in Ethiopia are among the highest in the world, the Ethiopian government developed an initiative focused on building multi-storied housing units organized into condominiums (22).

This study aimed to develop an interdisciplinary methodological approach integrating architecture, landscape urbanism, medical anthropology, and entomology to characterize exposure to malaria vectors in three condominiums in Jimma (23). Maps, drawings and sections were informed by ethnographic research and superimposed to entomological data to detect critical interactions between spatial factors (location and uses) and vector abundance and distribution in the selected condominiums. The extended results of this case have been published elsewhere (23). In this manuscript, we will focus on the specific use of drawings in this study.

### Cross-scale data collection and analysis

In both cases, the selected districts and sites were localized through maps and data retrieved from official open-source datasets, libraries (e.g., GIS) and open-source aerial views (such as EarthExplorer, usgs.gov, and openstreetmap.org). When epidemiological (Case 1, Lima) or entomological (Case 2, Jimma) data provided indications about the areas and factors requiring further investigation, we conducted joint fieldwork involving social scientists and urbanists.

Four scales were used to collect, organize, visualize, and analyze data: XL, the territory; L, the city; M, the neighborhood; and S, the house, a methodology used by Office of Metropolitan Architecture (24) and a frequently used way of analyzing through drawing in urbanism (14).

#### XL-scale: The territory we inhabit

The idea behind this level of analysis is that “disease and landscapes are both essentially spatio-temporal phenomena—always changing, but linked by geography” (3). In the studied cases, the XL-scale relies on drawings aimed to understand and describe the geography, including the climate of the territory and its structural elements, such as the hydrological network, its vegetation, and the larger context of the urban landscape, its urban growth, the larger transportation networks, and other man-made manipulations of the territory. The drawings were used to locate the study sites within their particular landscapes. Sections and infographics were superimposed to provide additional information on elements influencing the environmental conditions of the territory and potentially affecting transmission dynamics.

#### L-scale: The city, its building culture, and health systems

The L-scale describes and analyzes the city. Drawings at this scale take a closer look at the evolution of the city as a process of urban growth in relation to the building culture. The L-scale zooms in on the complexity of the urban landscape. In urbanism and urban planning, this is the scale at which zoning plans that legalize (and quantify) land use are made. In public health, the city level is classically understood through its administrative units, where the provision of health services is organized and health data are gathered and monitored. Maps and sections were drawn to tailor the open-source maps to the built reality on site, and quantify urban characteristics such as household density and land use.

Given the different scales of the cities (11 million inhabitants in Lima vs. 240,000 inhabitants in Jimma), different approaches were taken. In the case of Lima, a mapping exercise on zoning and land use was carried out to gather fine-grained data on spatial variables derived from preliminary epidemiological findings. For each of the six selected districts, the land use was quantified and converted to bar graphs to facilitate relative comparison between districts. This quantitative exercise was carried out using QGIS and AutoCAD. In the first step, we retrieved GIS data from the Ministry of Environment (25) and the National Institute of Geography (26) and converted them to AutoCAD keeping vectors and scale. In the second step, the official zoning maps from the Metropolitan Institute of Planification, with the relevant uses of space within each district, were traced in AutoCAD; a manual operation in which each relevant category received a layer and a color. The data from the official maps (ranging from 2018 to 2021) was then cross-checked and updated using aerial views from USGS Earth Explorer and fieldwork observations. The third step involved the calculation of areas within each category in AutoCAD. To facilitate comparisons of each category between districts, the conversion of areas (km^2^) was then scaled to scalebars ranging from 0 to 100% in Adobe InDesign, calculated with a spreadsheet. For housing, we made calculations based on the number of inhabitants reported in the 2017 Lima Census (27).

In the case of Jimma, condominiums are located within small urban administrative units called “kebeles”. Factors potentially linked to vector abundance and distribution were mapped, such as proximity of water bodies and floodplains, roads and vegetation. An aerial view provided a closer look at the location of the condominiums within their immediate context. Maps were then matched with information about urbanization processes and adaptation of the built environment collected through desk reviews, as well as through fieldwork observations on site.

#### M-scale: The neighborhood, the social, and urban tissue

At the level of the neighborhood, we focused on the urban tissue, as it offers an effective framework for identifying and describing the physical characteristics that contribute to the general and historical character of cities (28). The urban tissue is usually studied through 400 × 400 meter samples that represent the urban morphology of a territory, composed by open and built spaces (29). These fabric samples allow for comparison between settlements, building cultures and their relation to the geography, revealing the inner workings that constitute the materiality of cities and their neighborhoods (13).

The M-scale represents elements of the built and social environments that are often not included in official datasets. In the cases studied, the different urban tissues, identified through aerial views, were complemented with sections describing the built environment, as well as social activities conducted in public and private spaces within each neighborhood.

#### S-scale: Housing typologies and spatial appropriations

As a last step in dissecting the city, housing conditions within the different neighborhoods were mapped. Here, we analyzed social and spatial factors possibly influencing disease transmission, as well as modifications in the use of the home space created to improve health or reducing the risk of exposure. We aimed to capture the spatial configurations in which domestic routines such as cooking, eating, sleeping and leisure take place, as well as the demands of income-generating activities associated with the home space. In both cases, plans and sections of housing typologies were drawn, and colored layers were used to indicate spatial modifications and uses of the space. These appropriations and reconfigurations were then reinterpreted in relation to transmission dynamics and coping mechanisms used by local families to deal with perceived risks. Drawings were developed based on observations and interviews from joint fieldwork by urbanism and social science researchers.

## Results

### Airborne diseases in Lima, Peru (case 1)

#### XL – localizing Lima – map and section

**Figure 2:**
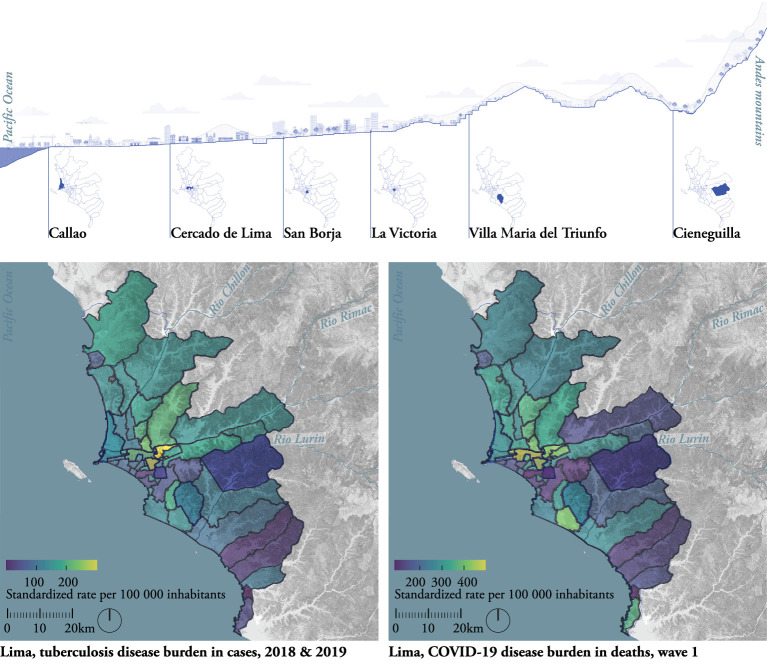
Lima, XL-scale. Map of the territory with topography, hydrology, and disease burden for TB **(Bottom left)** and COVID-19 **(Bottom right)** in the 50 districts of Lima A cross section from the ocean to the Andes, localizes the six selected districts **(top)** in relation to the geography. Drawn by the authors based on fieldwork observations, publicly available data (19, 25–27) and data from the Peruvian TB control program (20).

The maps in Figure 2 (Supplementary Figure 1) overlay the burden of TB and COVID-19 on an urban territorial map that includes topography, urbanization and water networks.

The maps indicate epidemiological observations but keep them tied to their location. They suggest an overlap in TB and COVID-19 disease burden. The disease distribution was heterogeneous across the districts: some of the centrally located districts such as Cercado de Lima and La Victoria (in yellow) had a relatively high disease burden, both for TB and COVID-19, while San Borja and Cieneguilla (in purple) had a relatively low burden.

The ocean-to-Andes section (Figure 2, top) locates the six focus districts and illustrates the geographical and climatological conditions of Lima. As Lima is located in a dry climate zone, rainfall is very low throughout the year (30) and the cold sea surface generates a sea fog covering the city, responsible for its high humidity (30). Moreover, the Andean mountains near the coastline block on- and offshore winds, potentially influencing humidity, cross-ventilation, and air pollution. The section also illustrates the diverse morphology of the city within the landscape, which impacts the distribution of the population across districts.

These observations give insights into Lima's urbanization process. The city is located on territory occupied in ancient times, which was colonized in the sixteenth century by Spanish explorers. They founded the city inland along the Rímac River, constituting what is today known as the historical center (e.g., Cercado de Lima) (31). Callao, located at the Rímac River, was subsequently established as the principal harbor of the city, later being transformed into Lima's main industrial area. The city developed in a “chessboard” pattern, a Spanish model of urbanization made with large avenues and square building blocks (“manzanas”) (31). After gaining independence from Spain, the city of Lima experienced accelerated growth during the twentieth century, resulting in suburbs and residential areas being built on flat surfaces (e.g., San Borja) and self-built neighborhoods sloping up to hills (e.g., La Victoria and Villa María del Triunfo).

#### L – Lima's districts – map and scalebar

**Figure 3:**
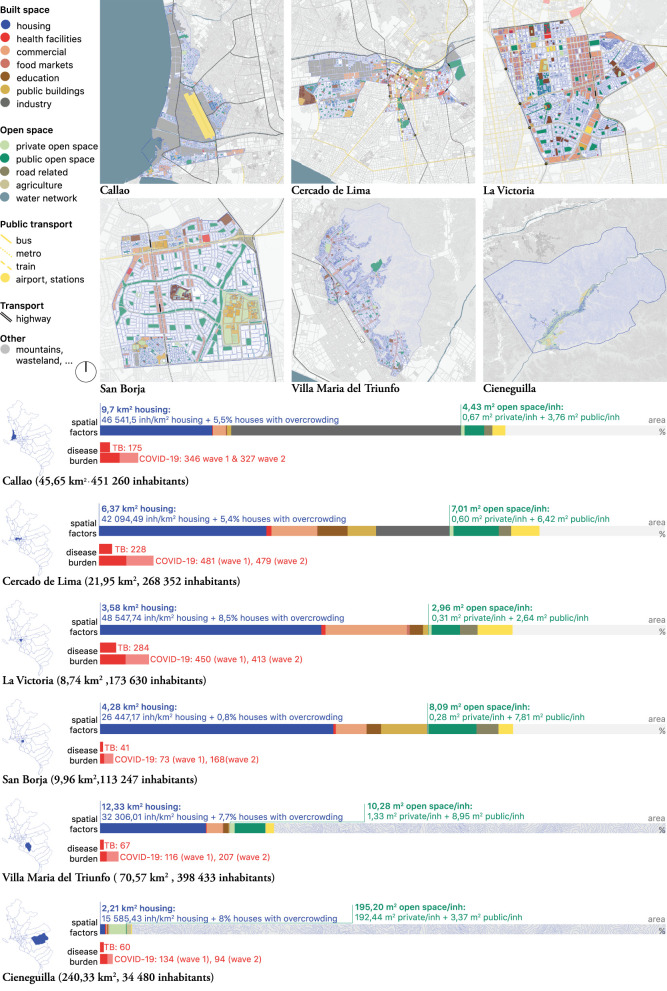
Lima, L-scale. Maps and bar-graphs of the “land use” were compiled to compare with the disease burden (per 100 000 inhabitants). Drawn by the authors based on fieldwork observations and publicly available data (25–27, 32–39).

Preliminary epidemiological analyses found associations between disease burden and socio-spatial factors such as ‘overcrowding’ [“percentage of households with more than 3.4 people per room, not including: bathroom, kitchen, hallway, garage”, reported through the national census (27)] and ‘urban green space’ [“the area of urban green per inhabitant”, self-reported per district (32)]. For the six selected districts, we studied these factors in more detail. We mapped and quantified the open and built space in layers by working *via* zoning plans, areal views, and fieldwork (Figure 3 and Supplementary Figure 2).

The mapping exercise added precision to the association between “overcrowding”, or household density, and TB/COVID-19 burden. Residential areas were mapped (Figure 3, blue layer) as preliminary epidemiological findings indicated that areas with higher average “household density” (not to be confused with “housing density”) registered more COVID-19 infections. The housing focus was also relevant considering that stay-home-orders were an important part of the COVID-19 mitigation strategy during the first wave.

Additionally mapped potential risk factors included spaces likely to be shared for a longer period of time, or to face overcrowding and ventilation issues, such as commercial areas, food markets, education facilities, public buildings, industry, and public transportation hubs. Health facilities were mapped because they may help to understand health seeking itineraries both within and beyond district boundaries.

The quantification of these socio-spatial factors was visualized using scale bars (Figure 3). The scale bars facilitated a quantitative comparison between districts and the assessment of possible associations between spatial factors and disease burden. Based on the comparison between these six districts, three emergent themes were further explored:

##### Nuancing “overcrowding”: On household density and inhabitants per km^2^ housing

Preliminary epidemiological findings associated higher household densities with a higher disease burden. A comparison of the maps with the blue housing layer (Figure 3) revealed a more nuanced reality for this preliminary association: of the six selected districts with the largest percentages of household density, only one (La Victoria) was a high-burden district for TB and COVID-19. We noticed that the three districts with the highest disease burden also have the highest number of inhabitants per km^2^ housing. These districts were La Victoria (48,547.74 inh/km^2^ housing), Callao (46,541.5 inh/km^2^ housing), and Cercado de Lima (42,094.49 inh/km^2^ housing). Several hypotheses arose: (1) residential areas in these districts were too small to house their citizens in times of lockdown, limiting social distance and ventilation to reduce risk of transmission; (2) the quality of housing in these districts limited the residents' ability to follow stay-home policies and isolation practices. Ethnographical and architectural research on uses of public space (M-scale), housing conditions (S-scale), and domestic practices (S-scale) tried to address these emerging themes.

The housing layer of the L-scale maps also provided insight into the different forms of residential areas. We saw that many inhabitants of La Victoria, for example, live on rocky hillsides (e.g., Cerro San Cosme), in densely populated forms of housing, while other neighborhoods in the same district expand over plain terrains, organized in “chessboard” grids, with large amounts of green space, and low housing densities. This raised the question whether homogenous policy measures aimed at mitigating the risk of infection have the expected outcome in such heterogeneous housing contexts? The L-scale maps helped to interpret the required urban conditions for the feasible implementation of these types of measures.

##### Nuancing “green space”: On the availability of public and private open space

San Borja and Villa Maria del Triunfo have a relatively high number of inhabitants per km^2^ housing but are characterized by large amounts of open space: San Borja (8.02 m^2^/inhabitant, of which 7.81 m^2^ public) and Villa Maria del Triunfo (10.28 m^2^/inhabitant, of which 8.95m^2^ public), in contrast to La Victoria (2.96 m^2^/inhabitant), Callao (4.43 m^2^/inhabitant) and Cercado de Lima (7.01 m^2^/inhabitant). Their disease burden was also significantly lower than that of La Victoria, Callao and Cercado de Lima. Cieneguilla, having the largest amount of open space (most of it privately owned), had the lowest disease burden. While concluding that open space influences disease burden would be too straightforward, these findings suggested that accessible open public spaces might mitigate disease burden in districts with large numbers of inhabitants per km^2^ of housing.

##### Other factors: Transportation lines and commercial areas

The XL maps indicated that the high-burden districts for TB and COVID-19, Cercado de Lima and La Victoria, are located in the center of the city. However, of the other mapped spatial factors, two more could potentially have a relation with the pattern of disease burden: Cercado de Lima and La Victoria are also the districts with the more public transportation lines and hubs (Figure 3, scalebar, yellow), as well as large commercial areas (Figure 3, scalebar, pink), both forms of urban infrastructure in which shared time and proximity are high.

#### M – the district's neighborhoods – section and plan

**Figure 4:**
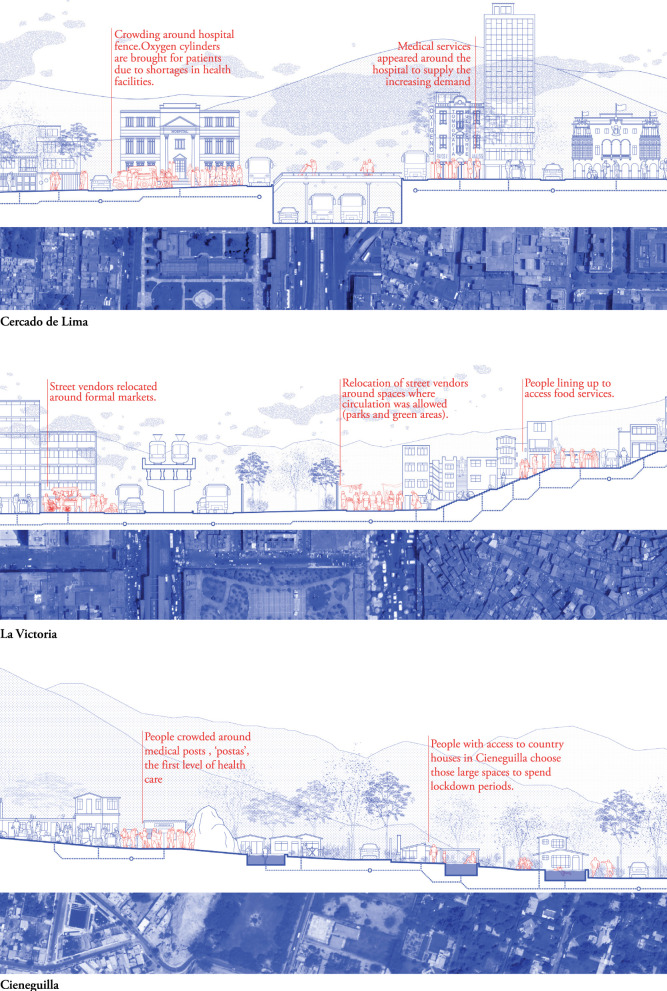
Lima, M-scale. Neighborhood section and collage map indicating the type of urban tissue for Cercado de Lima **(Top)**, La Victoria **(Middle)**, and Cieneguilla **(Bottom)**. Drawn by the authors based on fieldwork observations and publicly available data (40).

Analysis at the city and district levels informed further explorations at the next level, i.e., the neighborhood. The M-scale (Figure 4 and Supplementary Figure 3) focused on “overcrowding” and “open space” and included additional factors identified and mapped at the L-scale A collage-plan of urban tissues (Figure 4, bottom blue) that connects the section to the drawing on the ground (Figure 4, top) facilitates comparison between districts made out of similar tissues. The Spanish colonial chessboard tissue, with wide streets and square urban blocks with buildings, for example, is found in La Victoria, Cercado de Lima, and in other major districts located in the city center. The section illustrates the availability of and accessibility to public open space, but also describes the space (in blue) and its' uses (in red) before and during the pandemic. At this level, we captured how the nature of the districts' built environment allowed or hindered the inhabitants in adhering to control measures and illustrated the use of space around health facilities such as hospitals, primary care facilities located at the community level and private health facilities.

Figure 4 shows three sections in different neighborhoods of the central districts of Cercado de Lima and La Victoria, and compares them with Cieneguilla, known for its residential and recreational character. Districts with very diverse urban tissues, housing a wide variety of urban functions, such as Cercado de Lima and La Victoria, were forced to tailor the control measures to the heterogenous urban reality within the district in multiple sectors, including public offices, health facilities, tourism, and education. This contrasts with residential and low-density areas such as Cieneguilla that received more moderate impacts. These drawings nuanced the preliminary findings at previous scales:

##### Nuancing “overcrowding”: Housing conditions

The wide range of housing conditions found within and beyond district boundaries are illustrated in Figure 4. For example, inhabitants from the central districts moved to Cieneguilla during the lockdown, where they had second homes on the foothills, which offered larger and more open space (many homes had gardens and pools). These residences are, however, also supported by service staff who live in more crowded housing conditions on the fringes of the arid mountains (bottom section, left). Similarly, the nature of the crowded housing conditions in La Victoria (middle section) appear to differ from residential areas in Cercado de Lima (top section).

##### Nuancing “open public space”: Dynamics observed in public space

Qualitative data and fieldwork observations provided data for the red layer, indicating altered use of space during the pandemic. The drawings depict queues to access markets and health facilities, as well as emerging services (such as commerce in oxygen tanks outside hospitals). Reappropriations of public spaces, such as the rise of food markets in San Borja and relocation of commercial vendors around markets in La Victoria were also observed. All these cases exemplified the dynamic nature and adaptive capacity of public spaces in relation to social realities.

#### S – the housing unit – plan and section

**Figure 5:**
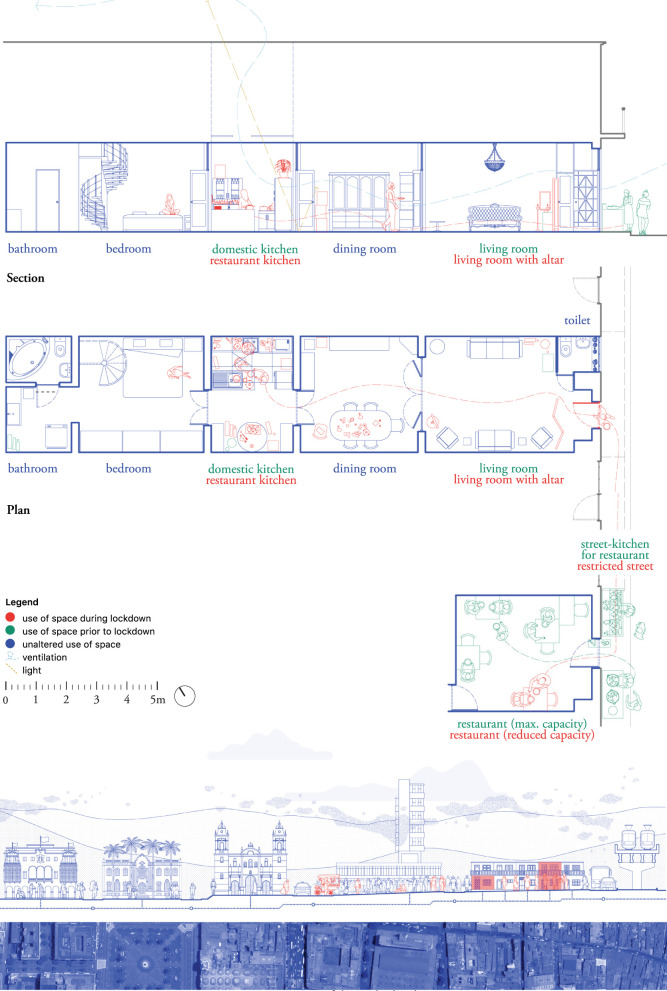
Lima, S-scale. Kitchen house. Drawn by the authors based on fieldwork observations.

The S-scale (Figure 5 and Supplementary Figure 4) was fundamental in our analysis, as it allowed a close look at social routines in interaction with the home space, based on both ethnographic and architectural data. The pre-pandemic home situation was drawn as the basis (blue), with an addition of altered uses of space during isolation and lockdown periods (red). In addition, close attention was paid to (private) open space in the immediate vicinity of the house, the amount of people populating the house (e.g., overcrowding), ventilation and the degree of light in the house, as critical elements in TB containment efforts. The drastic changes imposed by the pandemic are illustrated in Figure 5.

The case drawn in Figure 5 describes a family home composed of a living room, a self-constructed kitchen (that used to be a patio or inner yard), a bedroom, a laundry, two bathrooms (one for visitors at the front and one private at the back), and a small storage area on the roof (not drawn) accessible *via* a spiral staircase, all organized on one level. Ventilation within the home space was limited, as the only available window was closed in the construction of the visitors' bathroom. To increase ventilation during the pandemic, the front door remained open for most of the day. A skylight above the self-constructed kitchen enhanced ventilation and daylight. The housing space also included a small restaurant further down the street, where residents served and sold fried fish.

Pandemic restrictions included the restricted use of public space. To comply with the restrictions, the inhabitants decided to reorganize the restaurant, relocating the frying part of the process to the household kitchen (housing plan, red), using the pavement as an extension of the restaurant kitchen (blue and green layer), and serving and selling the fish at the restaurant. The whole process gave rise to a circulation pattern between the domestic kitchen and the restaurant that remained active during the months following the strictest lockdown. Other changes in the use of domestic space involved the construction of an altar at the entrance of the house to remember a relative who had passed away. The only bedroom of the house was used for isolation when household members were infected with COVID-19.

Through this level of analysis, we were able to identify close links between the home and public areas, as well as flexible demarcations separating both. Acknowledging this constant reconfiguration of domestic and economic routines in relation to the space was an important consideration when studying the routes of transmission of COVID-19 and TB, as it illustrated how the risk of exposure is constantly changing.

### Vector-borne diseases in Jimma, Ethiopia (case 2)

#### XL – localizing Jimma – map and section

**Figure 6:**
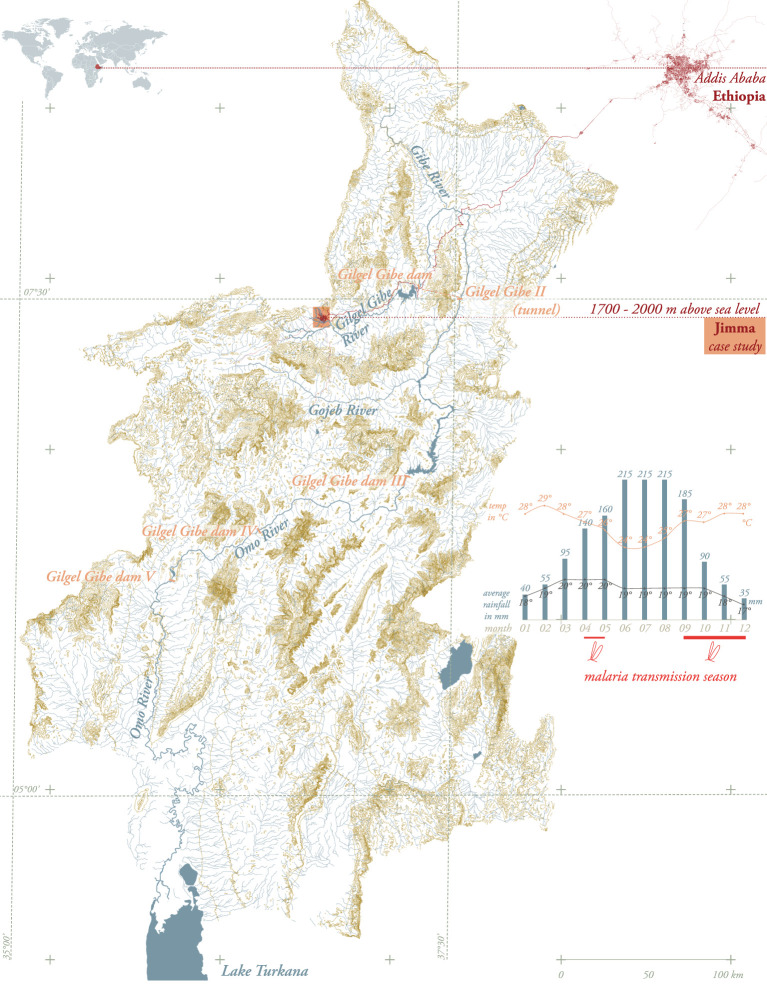
Jimma, XL-scale. Omo River basin including hydraulic manipulations such as dams. Drawn by the authors based on public available data (23).

A map (Figure 6 and Supplementary Figure 5) was drawn to describe the geographical conditions under which malaria transmission occurs in Jimma. The map was made developed to localize spatial factors known to be involved in transmission, such as altitude, climate, and hydrology.

Jimma is located at 1,700–2,020 m above sea level, along the Gilgel Gibe River, in the Omo-Gibe basin, and is part of the highland fringe areas (1,500–2,500 m) affected by frequent malaria epidemics (21). Transmission is seasonal and largely unstable. The major transmission period follows the June to September rains and occurs between September and December, while a minor transmission season occurs between April and May, following the February and March Belg rains (21). The major transmission season occurs in almost every part of the country. The map in Figure 6 draws the hydrology of the Omo River basin—including hydraulic manipulations such as dams—as an overview of artificial and natural water bodies can help to gain a more comprehensive understanding of systemic interactions potentially linked to generation of mosquito breeding sites.

#### L – the city of Jimma – map

**Figure 7:**
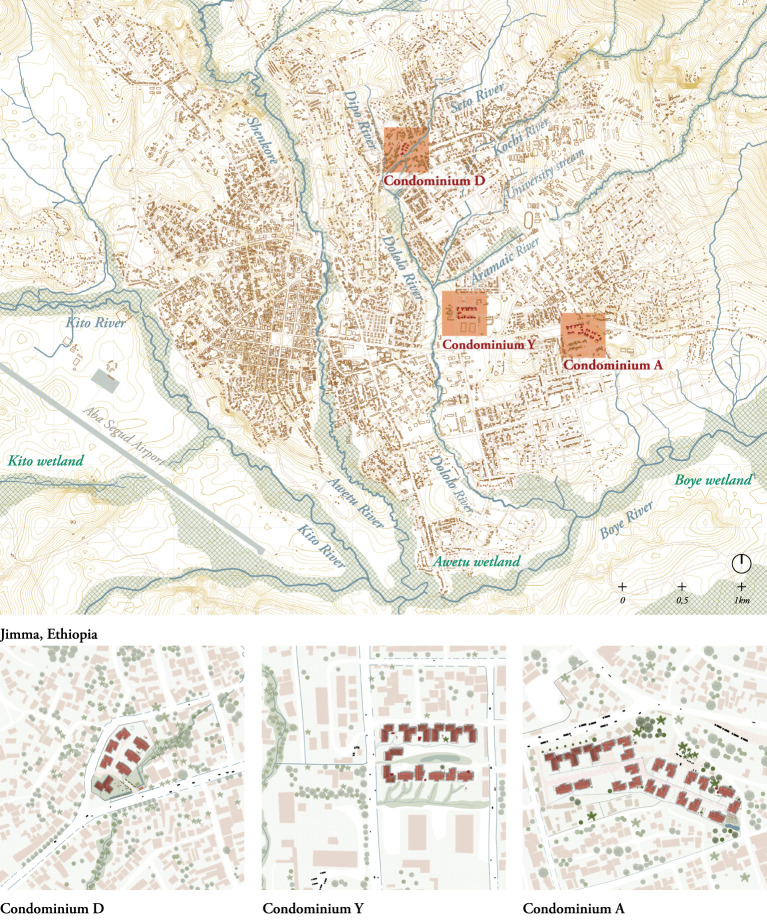
Jimma, L-scale. **(Top)**: The map shows the water network in the dry season, with floodplains at its narrowest (23). **(Bottom)**: zoom of the three researched cases. Drawn by the authors based on field work observations.

The L-scale map (Figure 7 and Supplementary Figure 6) localized and characterized the three condominiums researched. From this perspective, interactions between factors in the natural and the built environment of the condominiums were identified. The map located the city of Jimma in a mountainous landscape characterized by an extended water (blue) and wetland (green) network in the south and west (note that the map depicted the dry and not the rainy season situation with larger floodplains) and densely forested hills in the north.

Condominium D was built on the floodplain of the Dipo River, with the most southern housing units prone to permanent flooding during the rainy season. On site, the river was canalized but the canal was under-dimensioned, which has generated a permanent swampy site directly around the canal even in the dry season. At the moment of the study, Condominium D had 246 residents divided into 90 households built under the M-1, M-2 and T-9 housing typologies. Condominium Y was located on wet, low-laying lands and houses 1,512 residents in 378 households. Condominium A, in contrast, was a road-based condominium that hosted 1,272 residents organized into 318 households. Condominium A was the least regulated of the observed sites, with no fences or physical barriers delimiting its boundaries. It had a wet, low-laying area holding solid waste at the lowest point of the site, fed by a water spring located in the center of the common area; it was also characterized by many informal housing extensions at the ground level.

The drawings were a snapshot in time. As such, they revealed another perspective on the city's process of swift urbanization, as it located the condominiums on low-lying sites that are part of the different river networks. With mountainous landscape in the north, and rivers in the south, the footprint of the city is reaching its geographical limits and urbanization is becoming a process of densification rather than expansion. In the case of Jimma, this means that the condominiums, built by the government and sold to private owners per unit, are located on publicly available and low-lying land that is often found unfit for privatized real estate development.

#### M – the condominium neighborhood – plan and section

**Figure 8:**
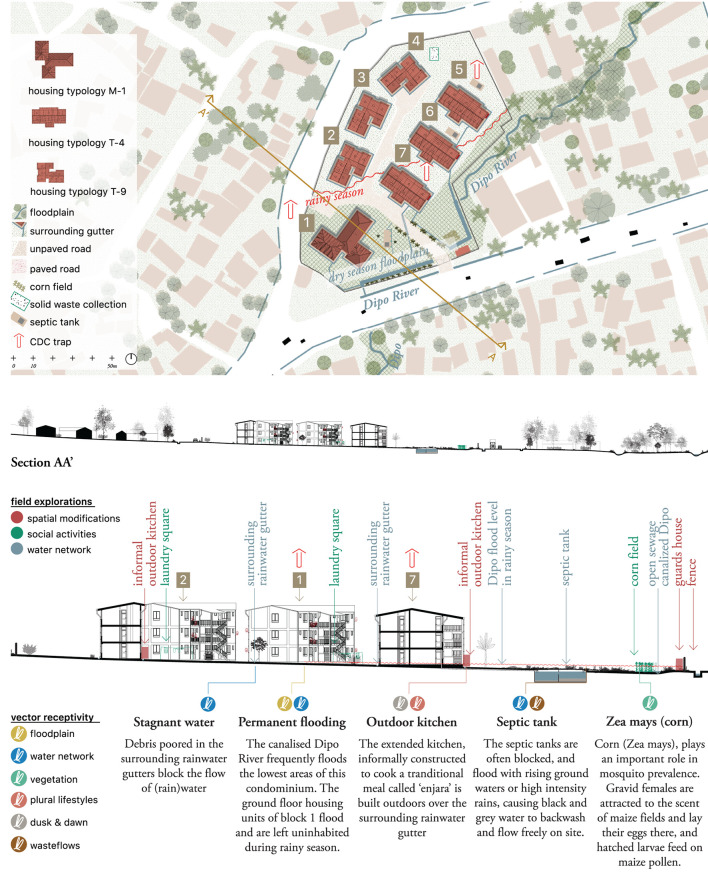
Jimma, M-scale. Condominium D. Drawn by the authors based on fieldwork observations and publicly available data (23).

Each condominium was mapped in terms of plan and section at neighborhood scale using layers (Figure 8 and Supplementary Figure 7). Structural and non-structural spatial modifications were mapped in red, including informal housing extensions, additional doors, removed walls, ground floor elevations and windows' adjustments. Uses of the space linked to sociocultural activities, such as cooking, laundering or growing crops were indicated in green. These observations formed a layer of appropriation of either the condominiums' common spaces or the standardized housing plan. Finally, a blue layer highlighted the rain, black and gray water networks, including rivers, canals, septic tanks, rainwater gutters and water taps.

In this case, it was at the M-scale (Figure 8) that integration with quantitative (entomological) data took place. The drawing showed the blocks where mosquito traps were placed and where data were collected. Accordingly, six layers of possible breeding sites for vectors were identified (see section, bottom): swampy conditions due to constructing on the floodplains (yellow); stagnant water due to blockage of the water network (blue); the presence of vegetation (green); the appropriation of space to dwelling cultures (pink); exposure at dusk and dawn (gray); and malfunctioning waste flows (brown).

As an example, we can zoom in on the vegetation layer (green). Jimma is known for rich diversity of vegetation in the city and on the mountain slopes. In the Boye Wetlands, species such as *Typha latifolia, Cyperus latifolius* and *Cyperus rotundus* are found. In the remaining areas, tree species such as the Eucalyptus, Cypress and exotic Grevillea are common, along with bush and shrub vegetation. However, due to the leveling of the site for building purposes, the land was flattened and vegetation was completely erased. Consequently, none of these species are found on site. The design of the condominium sites also does not show predefined or planned vegetation, focusing only on paved surfaces and open grass fields. The map and section indeed illustrate a condominium without vegetation, forming a clear discontinuity in Jimma's green landscape. The lack of planned green spaces is compensated for by the residents, who have individual informally claimed gardening areas, which bring potential mosquito breeding sites into the immediate vicinity of the households.

The use of qualitative and entomological data at this level allowed for a clearer understanding of the physical context of the condominium. In the case of Condominium D, for example, we were able to demarcate the extent of the rainy season floodplain footprint guided by the explanations of residents interviewed for this study. Similarly, the entomological data collected by placing CDC traps on different floors and various areas of each condominium facilitated the selection of areas for which additional data was required (ground floors in relation to other areas). At the M-scale, the neighborhood drawings illustrated how location, topography and water and waste networks influenced possible vector presence close to the domestic unit.

#### S – the housing unit – plan and section

**Figure 9:**
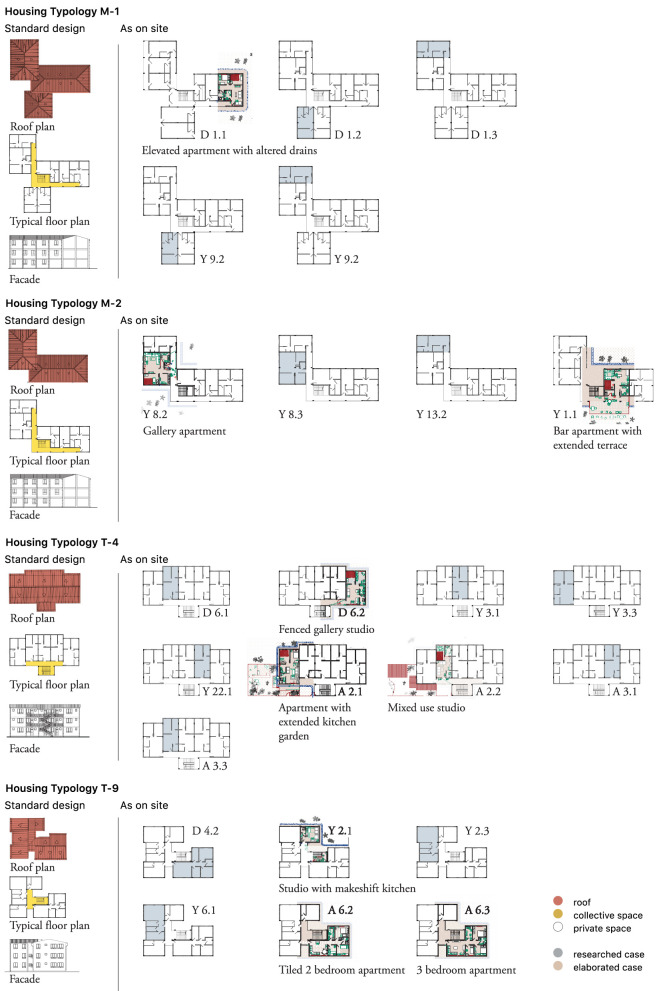
Jimma, S-scale. Standard housing typologies (M-1, M-2, T-4, and T-9), their standard design plans (roof plan, typical floor plan, and façade) and their modifications as observed on site. Joint fieldwork (social sciences and urbanism) was carried out in 24 housing units dispersed across the three condominiums. A selection of nine representative typologies is further elaborated. Drawn by the authors based on fieldwork observations.

**Figure 10:**
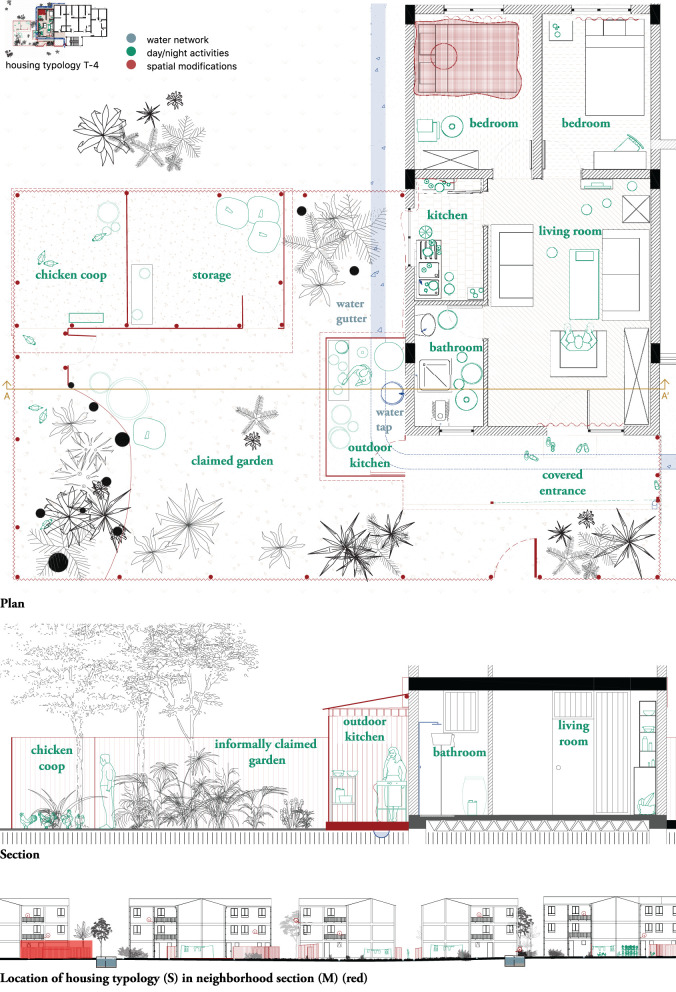
Jimma, S-scale. Housing typology T-4 in Condominium A. Ground floor apartment with extended kitchen garden. Drawn by the authors based on fieldwork observations (23).

To gain a better understanding of the contribution of domestic space and daily routines to vector-borne disease transmission, as well as adaptation mechanisms displayed by residents, housing typologies were drawn (see Figures 9, 10). Each of the housing typologies researched (24 in total) was drawn in terms of plan and section (Figure 9 and Supplementary Figure 8). Nine typologies were further elaborated, as they were found to be representative of the circumstances found in all 24 housing units. The color-coded layers used in the M-scale were also used here, in Figure 10 (Supplementary Figure 9), indicating spatial modifications (red), social activities during the day and night (green) and the water network in the dry and the rainy seasons (blue).

The typologies were not mapped as discrete objects (in accordance with their design as standardized models) but were related each time to their specific location on site in the neighborhood. The drawing incorporated information from both the interviews and the spatial observations.

The plan and section of the ground floor apartment with extended kitchen indicated the indoor use of square bed nets, as well as fine-grained curtains in front of every window and the front door. The drawings also illustrated how the standard model of T-4 was informally extended by the residents (in this case a five-member family), with an outdoor kitchen and a garden added (both drawn in red). The outdoor kitchen was built to meet the inhabitants need to cook “Injera”, a traditional pancake made of fermented teff flour and cooked on a charcoal or firewood open fire, a tradition impossible to execute indoors. The outdoor kitchen had a ventilated roof, a light bulb and was constructed on top of the rainwater gutter, potentially exposing those who cook to the stagnant water and other environmental conditions. The informally claimed garden sheltered a chicken coop, an extra storage space and grew a wide variety of vegetation.

The drawings in this case illustrated actual situations on site, unveiling a spatial complexity of vector presence as linked to social and spatial habits. They also suggested articulations between factors potentially linked to the emergence of mosquito breeding sites in each one of the spatial contexts studied.

## Discussion

**Figure 11:**
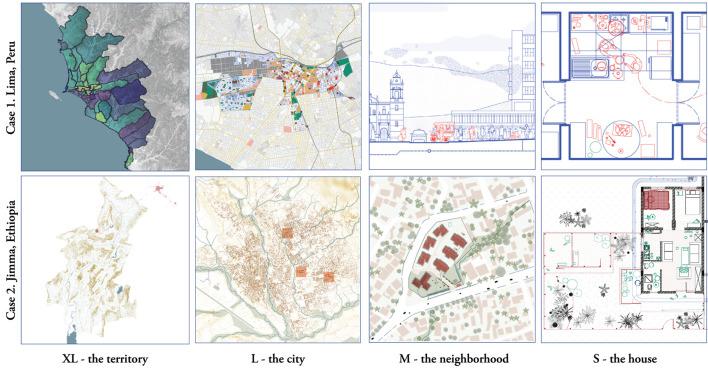
XL to S. Examples of drawings produced at each scale in the studied cases. Drawn by the authors.

The drawings made for Lima (Figures 2–5) and Jimma (Figures 6–10) explicitly revealed geographies and spatial relationships that often remain implicit in global health research. Each of the drawings helped contextualizing and adding complexity to spatial variables commonly approached in terms of geographical boundaries or simply as “proxies” to disease exposure. While constantly intertwining macro and micro scales, drawing emerges a process that differentiates and connects a sequence of spatial factors to entomological, epidemiological and social factors potentially impacting disease transmission at each level (Figure 11). The layered drawings, as analytical instruments, aid to conceive space as a dynamic variable that is built and transformed in interaction with the climate and geography of the territory (XL), its building and housing cultures (L, M, S), the economy and policy (L, M) and ultimately, the cities' epidemic history. These fine insights could be translated into tailored, context-specific at each level of preparedness in urban contexts.

The drawing processes and tools presented here helped us to reimagine space in interdisciplinary health research in three specific ways:

### Drawing to identify systemic relations

Oscillating between the multiple scales of the city, drawing allows for the spatial dissection of the heterogenous patterns of disease distribution in urban contexts. The presented drawings link geographical conditions and building cultures to disease distribution patterns, but also to uses of the space affecting the adoption of prevention practices. They provide a structured analysis of the urban context, visualizing relations between spatial, social and biomedical systems. In the Ethiopian case, the drawings revealed variations in the impact of socio-spatial factors, such as the proximity to river network with its floodplain, forests and agricultural conditions, as well as waste (water) networks with importance on vector-borne disease. Drawings related to airborne diseases, as was the case in Lima, selected different layers and focused on the availability of health services such as hospitals, as well as open spaces, commercial areas and places of crowding. The combination of these layers with data from different disciplines can help reveal the social and physical conditions of health.

Risk of exposure was also analyzed across scales. In Jimma, the geography defines the territory as malaria-prone, and the fact that most condominiums are built on low-lying land and floodplains creates favorable conditions for transmission. Domestic routines and spatial modifications to the house may be effective in reducing exposure but should always be considered in relation to the risk of infection at the level of the larger urban context. It is difficult to construct “malaria-proof” housing when living in a floodplain in malaria-prone geographies. In Lima, the sustained transmission of TB forced us to explore factors at all scales. Identifying the similar distribution of COVID-19 mortality and TB cases at the XL-scale was intriguing but not sufficient to explain burden of disease; a closer look at centrally located districts in relation to housing conditions and, more specifically, ventilation, daylight and household density or inhabitants per km^2^ housing seemed necessary to avoid simplistic attributions of risk.

### Drawing to bridge quantitative and qualitative research methods

Complementing quantitative (epidemiology for Lima, entomology for Jimma) and qualitative (ethnographic in both cases) data, the urbanist perspective and descriptive drawings proved to be instrumental in the synthesis of different forms of data. In both cases, the drawings bridged quantitative and qualitative research methods: when used in an iterative form, findings from the process of drawing fed back into the epidemiological research (e.g., the epidemiological model in Lima, L-scale), or refined research questions for ethnographic work (e.g., modification of water networks in Jimma, S-scale). In the cases presented, the drawings were conceived to localize and spatialize the multitude of information from all disciplines involved, aiming to facilitate interdisciplinary collaboration. They also helped to contrast secondary data with spatial realities on site. The mapping exercise on “urban green spaces” in Lima, for example, gave insight into the quality of the publicly available data. The quantification of “urban green spaces” was refined to “urban open spaces” as for airborne transmission, the idea that a public space is open and ventilated is more important than it being green (41, 42). The availability of open space was also linked to the discussion on accessibility to public and private open areas emerged from discussions with participants in ethnographic research. Similar to the Cholera Map of Broad Street, the drawings used in this study were instrumental in refining hypotheses and proposing alternative lines of thought.

### Drawing to inform urban and public health planning

In Lima, data concerning the disease burden of TB and COVID-19 was only available at the district level and did not reflect the social and urban heterogeneity found within the district boundaries. While COVID-19 measures were homogenous for all districts, their implementation varied according to the social and urban realities within each district. Drawings helped to explain the differentiated adoption of protective measures at different levels and indicated how factors related to the urban tissue of the districts, such as the amount of open space, the number of people housed in residential areas, household density and the density of transportation networks could have a relation with disease transmission. These drawings can inform urban planning and the design of strategic urban health projects.

Similarly, different mapping scales also relate to different operational stakeholders and decision-making parties. In Jimma, vector control strategies demand involvement of actors at different levels. This starts at the XL policy level (decision-making on where the condominiums are built), passes through condominium governance (when and how waste and water networks are connected, for instance), and goes down to the individual radius of action, deciding about taking vector-protective measures at the household level. In both cases, the drawings helped to visualize how policy measures could be implemented. Ultimately, these integrated data could also help tailor health policy measures to a heterogenous urban reality.

## Drawing a conclusion

In the two studies presented, different disciplines collaborated to better understand the heterogenous patterns of airborne and vectors borne disease distribution. Drawings from the field of urbanism are analytical instruments that, by crossing scales and integrating data from different disciplines (in research) and sectors (in governance), facilitate a systematic analysis of spatial variables at different levels. This paper has illustrated how drawings can be instrumental in unraveling the patterns of disease transmission in urban contexts. Through visualizing and locating the collected data, drawings support the formulation of alternative hypotheses and lines of thought, and, when used in an iterative research process, findings from the process of drawing could feed back information to the social sciences and epidemiological research. Drawings re-imagine space and are useful research tools to identify systemic relations, bridge quantitative and qualitative research methods, and eventually inform urban and public health planning.

### Limitations

The production of drawings in the cases hereby presented heavily relies on the availability of secondary data (for the XL and L scales), as well as on the quality of the primary data collected (for M and S scales). Quality and availability of information can constitute a significant limitation in this case. In Lima, for example, data was collected in November and December of 2021, when physical distancing measures were still in place. This limited our capacity to visit homes as well as to conduct extended observation in public areas, and as such, limited our capacity to recreate spatial realities onsite. Furthermore, it is important to acknowledge that maps of observed data (such as Figure 3) are insufficient to confirm geographical patterns of health outcomes. Further statistical analyses, focusing on revealing patterns at the XL scale, should account for space, time, and other covariates, a topic that will be addressed in a separate manuscript. Moreover, because epidemiological studies typically involve data that are aggregated at the level of administrative units, we should acknowledge a risk of bias related to the choice of the unit of observation (i.e., ecological fallacy and modifiable areal unit problem). In the case of Lima, this is particularly relevant for the epidemiological component where the district was the only possible unit of observation due to data availability, As part of larger interdisciplinary studies, drawings extend or illustrate analyses in other disciplines; therefore, early conceptualization of data integration processes can extend the possibilities of urbanism and its analytical tools in public health; however, the reach and scope of this integration is highly dependent on the nature of the studies being conducted, the disciplines involved and the availability of resources. As such, the information hereby provided for each case also accounts for the fact that Lima's study was exploratory, and Jimma's case was implemented through a pump priming scheme. Application of this methodology in long term studies is promising but remains to be fully explored. Finally, although the diversity of drawings included in the studies intends to increase accessibility to non-specialized publics, its production and interpretation demands specialized knowledge that can limit their usability in scientific and non-scientific scenarios.

## Data availability statement

Quantitative data supporting the conclusions of this article will be made available by the authors, without undue reservation. Qualitative data supporting the qualitative findings of this study are retained at the Institute of Tropical Medicine, Antwerp and will not be made openly accessible due to confidentiality concerns as the dataset cannot be fully anonymised. Data requests can be addressed to ITMresearchdataaccess@itg.be/.

## Ethics statement

The studies involving human participants were reviewed and approved by Case 1, Lima: The protocol was approved by the Ethics Committee at the Universidad Peruana Cayetano Heredia in Lima (SIDISI 205587) and by the Institutional Review Board of the Institute of Tropical Medicine in Antwerp. All participants interviewed went through the informed consent process and gave their consent before enrollment. We used the Bridging Research Integrity and Global Health Epidemiology (BRIDGE) guidelines to guide the conduct of this study (43). Throughout the study period, the investigators together with an external coach, Dr Sandra Alba, held seven online meetings to discuss the application of the BRIDGE guidelines to this study. Case 2, Jimma: All components of this study were reviewed and approved by the Institutional Review Board of the Institute of Tropical Medicine in Antwerp, Belgium (ref: 1241/18) and Addis Ababa University Addis Ababa University (ref: 081/19/2019). Oral informed consent was obtained from all research participants and the images altered to protect the residents' identity. Written informed consent for participation was not required for this study in accordance with the national legislation and the institutional requirements.

## Author contributions

SD: conceptualization, methodology, data curation, formal analysis, visualization, and writing (original draft preparation, review, and editing). CN-S: conceptualization, methodology, data curation, formal analysis, and writing (original draft preparation, review, and editing). MD: investigation, data curation, formal analysis, and visualization. TH: visualization. AH, KSola, JC, VV, KSolo, and DY: investigation, data curation, formal analysis, and writing and review of final manuscript. LO and KV: methodology, investigation, data curation, formal analysis, supervision, funding acquisition, and writing (original draft preparation, review, and editing). KG and MV: methodology, investigation, formal analysis, funding acquisition, and writing (original draft preparation, review, and editing). All authors contributed to the article and approved the submitted version.

## Funding

This work in Jimma, Ethiopia, was supported by pump-prime funding from the BOVA Network (Building Out Vector-borne diseases in sub-Saharan Africa). The work on Lima, Peru, was supported by the Belgian Directorate-general for Development Cooperation and Humanitarian Aid (DGD) *via* the fourth Framework Agreement (FA4) with the Institute of Tropical Medicine, Antwerp.

## Conflict of interest

Author TH was employed by Witteveen+Bos. The remaining authors declare that the research was conducted in the absence of any commercial or financial relationships that could be construed as a potential conflict of interest.

## Publisher's note

All claims expressed in this article are solely those of the authors and do not necessarily represent those of their affiliated organizations, or those of the publisher, the editors and the reviewers. Any product that may be evaluated in this article, or claim that may be made by its manufacturer, is not guaranteed or endorsed by the publisher.
