# Supplementary material for: Drawings as tools to (re)imagine space in interdisciplinary global health research
Source: Front Public Health. 2022 Dec 5;10:985430. doi: 10.3389/fpubh.2022.985430 (PMC9762521; doi:10.3389/fpubh.2022.985430)
Supplement: Supplementary file 1 [file Image_1.pdf]

Frontiers in Public Health
TYPE Methods
DOI 10.3389/fpubh.2022.985430

Drawings as tools to (re)imagine space in interdisciplinary global health research

2022 Stefanie Dens, Claudia Nieto-Sanchez, Mario De Los Santos, Thomas Hawer, Asgedom Haile, Karla Solari, Jesus Cisneros, Victor Vega, Kalkidan Solomon, Adamu Addissie, Delenasaw Yewhalaw,Larissa Otero, Koen Peeters Grietens, Kristien Verdonck and Maarten Van Acker

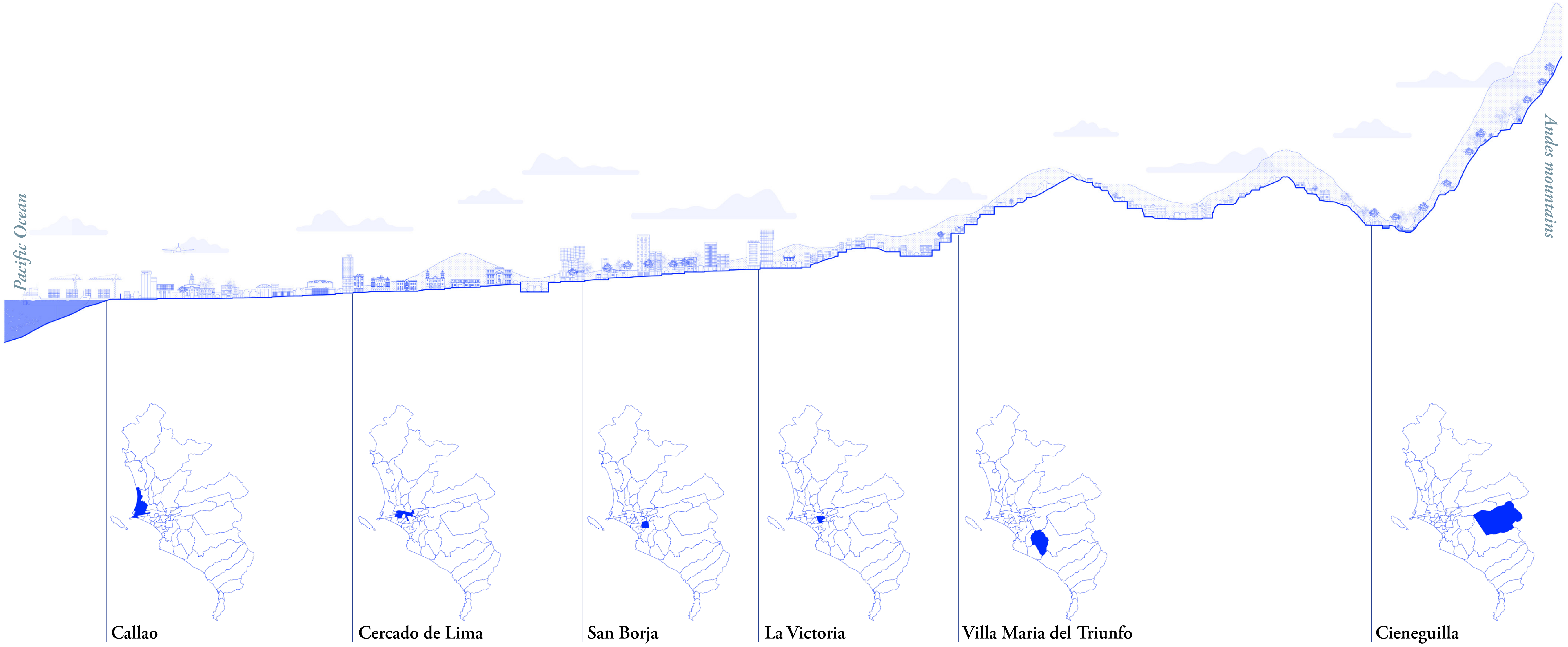

FIGURE 2 (top)
Lima, XL-scale.

A cross section from the ocean to the Andes, localizes the six selected districts in relation to the geography.

Correspondence:
Stefanie Dens
stefanie.dens@uantwerpen.be

This article was submitted to
Public Health Policy,
a section of the journal
Frontiers in Public Health

Frontiers in Public Health
TYPE Methods
DOI 10.3389/fpubh.2022.985430

Drawings as tools to (re)imagine space in interdisciplinary global health research

2022 Stefanie Dens, Claudia Nieto-Sanchez, Mario De Los Santos, Thomas Hawer, Asgedom Haile, Karla Solari, Jesus Cisneros, Victor Vega, Kalkidan Solomon, Adamu Addissie, Delenasaw Yewhalaw,Larissa Otero, Koen Peeters Grietens, Kristien Verdonck and Maarten Van Acker

FIGURE 2 (bottom) Lima, XL-scale.

Map of the territory with topography, hydrology, and disease burden for TB (left) and COVID-19 (right) in the 50 districts of Lima

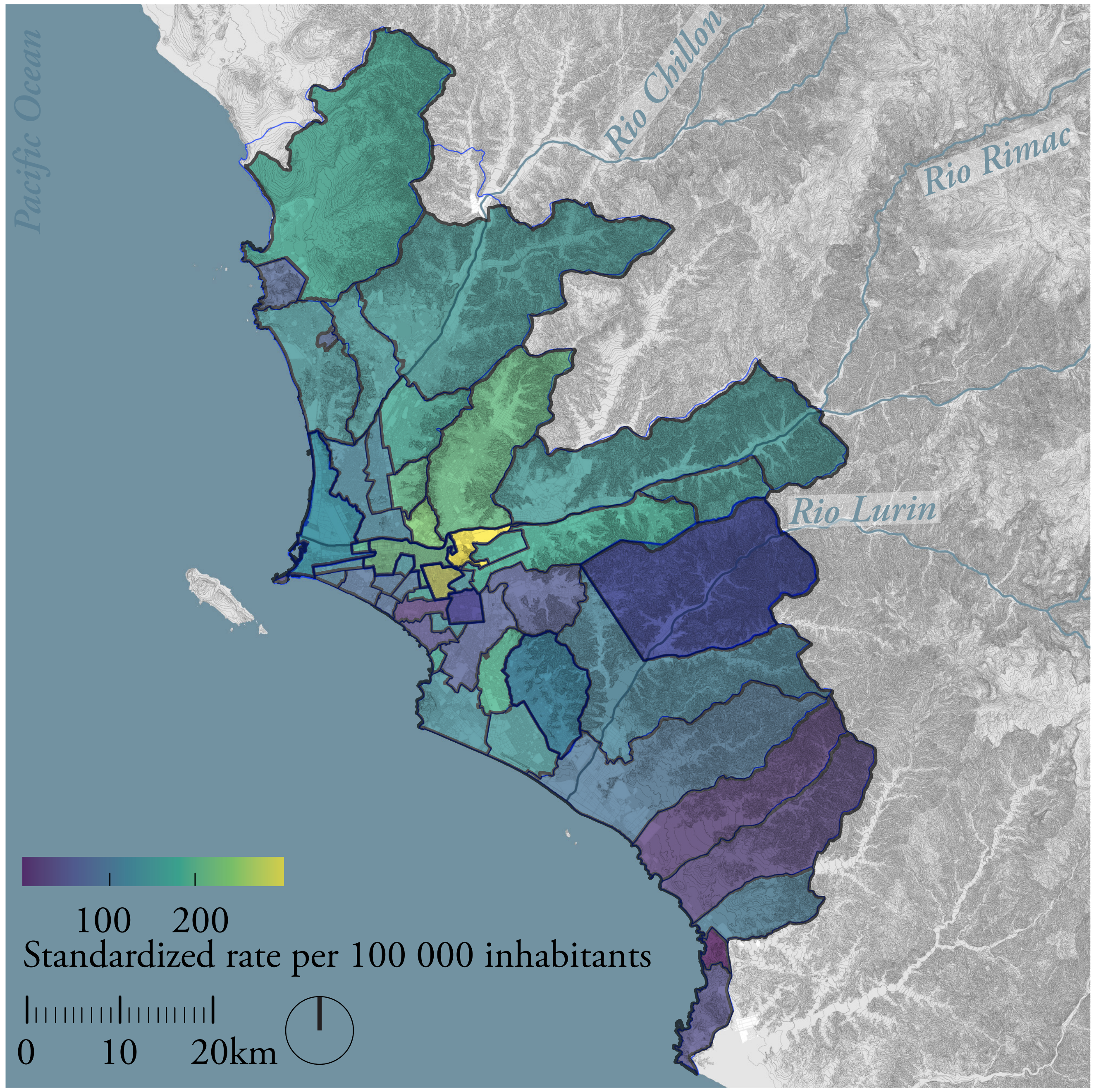

Lima, tuberculosis disease burden in cases, 2018 & 2019

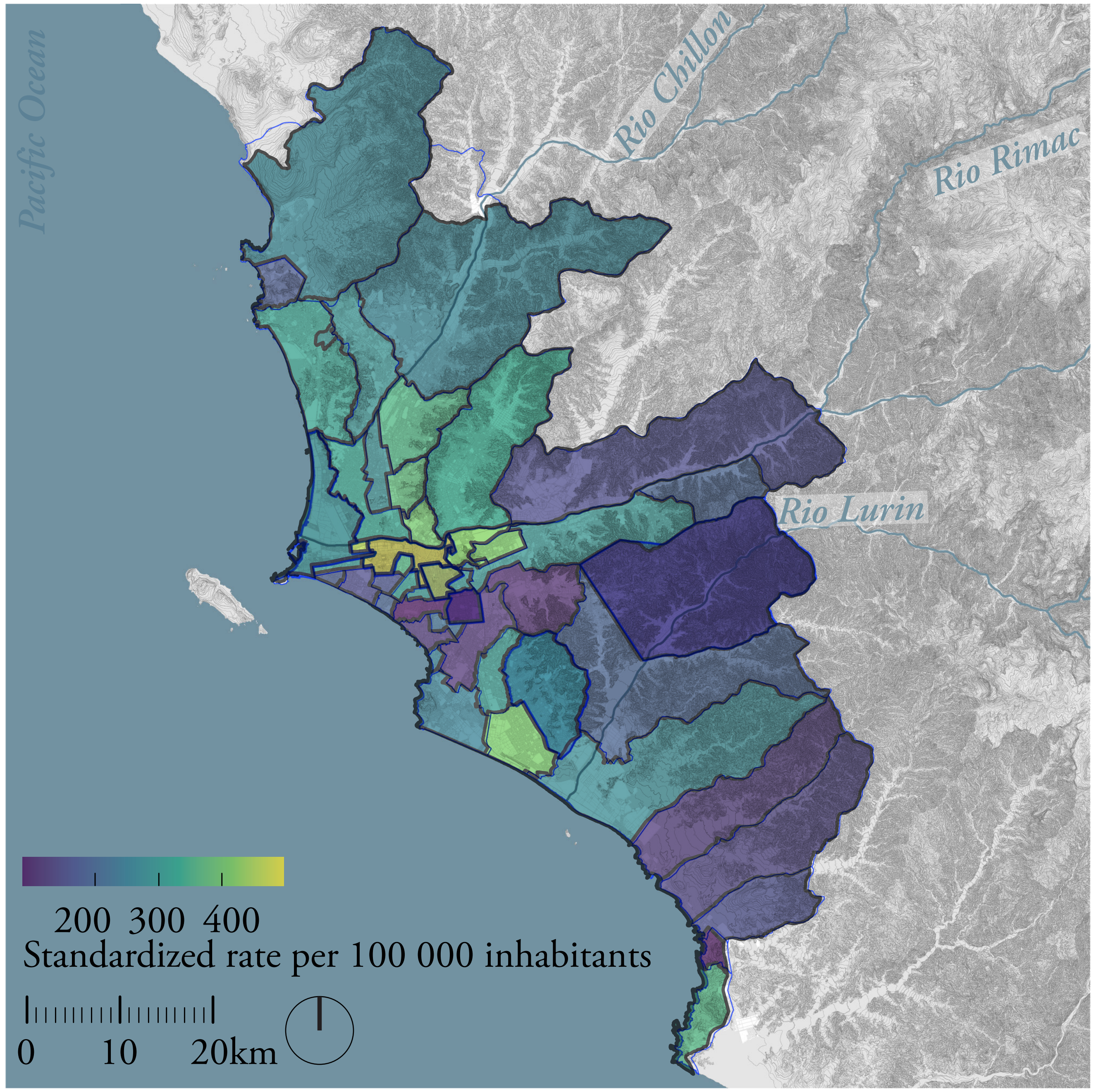

Lima, COVID-19 disease burden in deaths, wave 1

Correspondence:
Stefanie Dens
stefanie.dens@uantwerpen.be

This article was submitted to
Public Health Policy,
a section of the journal
Frontiers in Public Health
