# Supplementary material for: Drawings as tools to (re)imagine space in interdisciplinary global health research
Source: Front Public Health. 2022 Dec 5;10:985430. doi: 10.3389/fpubh.2022.985430 (PMC9762521; doi:10.3389/fpubh.2022.985430)
Supplement: Supplementary file 5 [file Image_5.pdf]

Drawings as tools to (re)imagine space in interdisciplinary global health research

2022 Stefanie Dens, Claudia Nieto-Sanchez, Mario De Los Santos, Thomas Hawer, Asgedom Haile, Karla Solari, Jesus Cisneros, Victor Vega, Kalkidan Solomon, Adamu Addissie, Delenasaw Yewhalaw,Larissa Otero, Koen Peeters Grietens, Kristien Verdonck and Maarten Van Acker

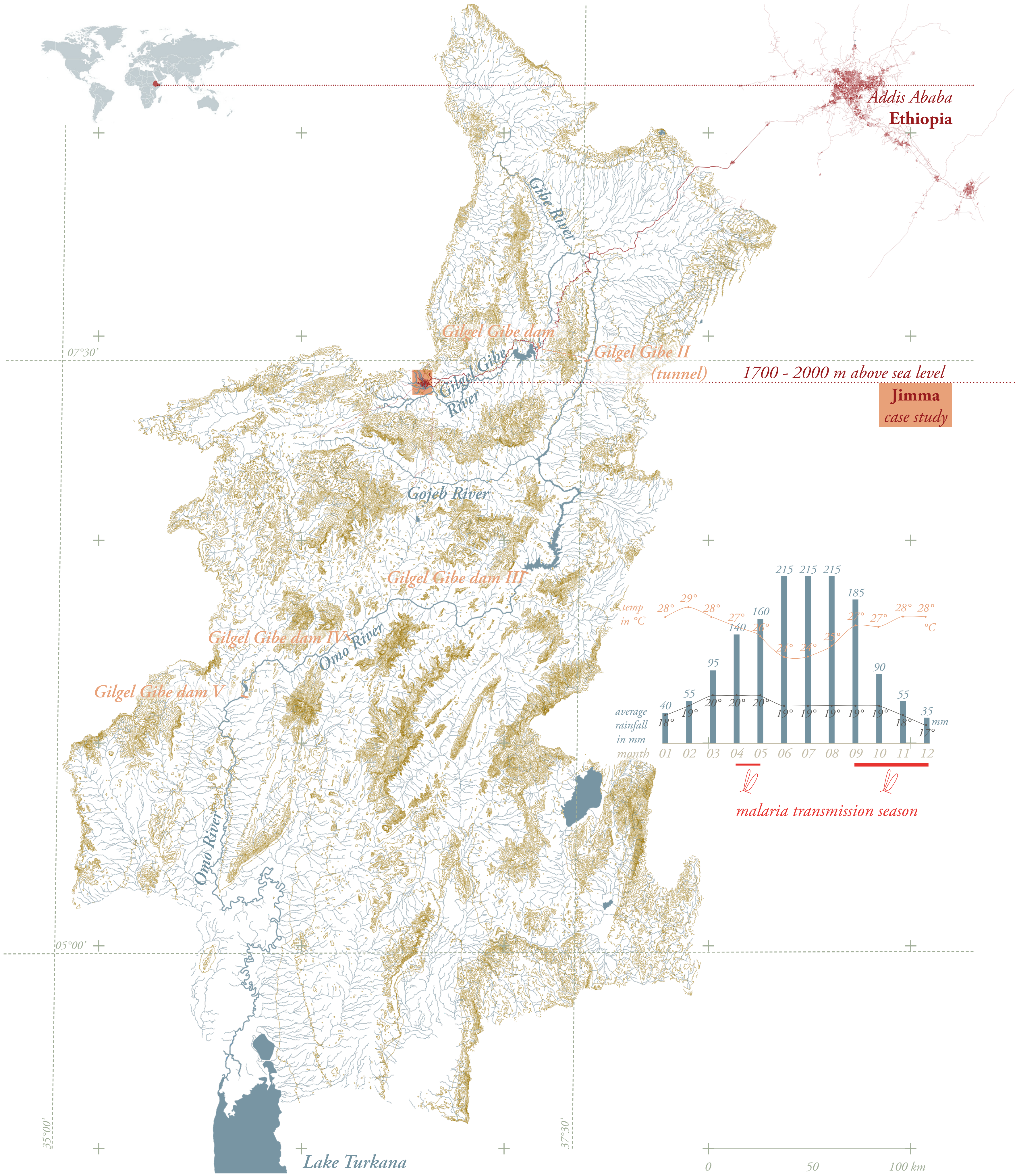

FIGURE 6
Jimma, XL-scale.

Omo River Basin including hydraulic manipulations such as dams.

Correspondence:
Stefanie Dens
stefanie.dens@uantwerpen.be

This article was submitted to
Public Health Policy,
a section of the journal
Frontiers in Public Health
