# Supplementary material for: Drawings as tools to (re)imagine space in interdisciplinary global health research
Source: Front Public Health. 2022 Dec 5;10:985430. doi: 10.3389/fpubh.2022.985430 (PMC9762521; doi:10.3389/fpubh.2022.985430)
Supplement: Supplementary file 7 [file Image_7.pdf]

Drawings as tools to (re)imagine space in interdisciplinary global health research

2022 Stefanie Dens, Claudia Nieto-Sanchez, Mario De Los Santos, Thomas Hawer, Asgedom Haile, Karla Solari, Jesus Cisneros, Victor Vega, Kalkidan Solomon, Adamu Addissie, Delenasaw Yewhalaw,Larissa Otero, Koen Peeters Grietens, Kristien Verdonck and Maarten Van Acker

FIGURE 8
Jimma, M-scale.

Condominium D

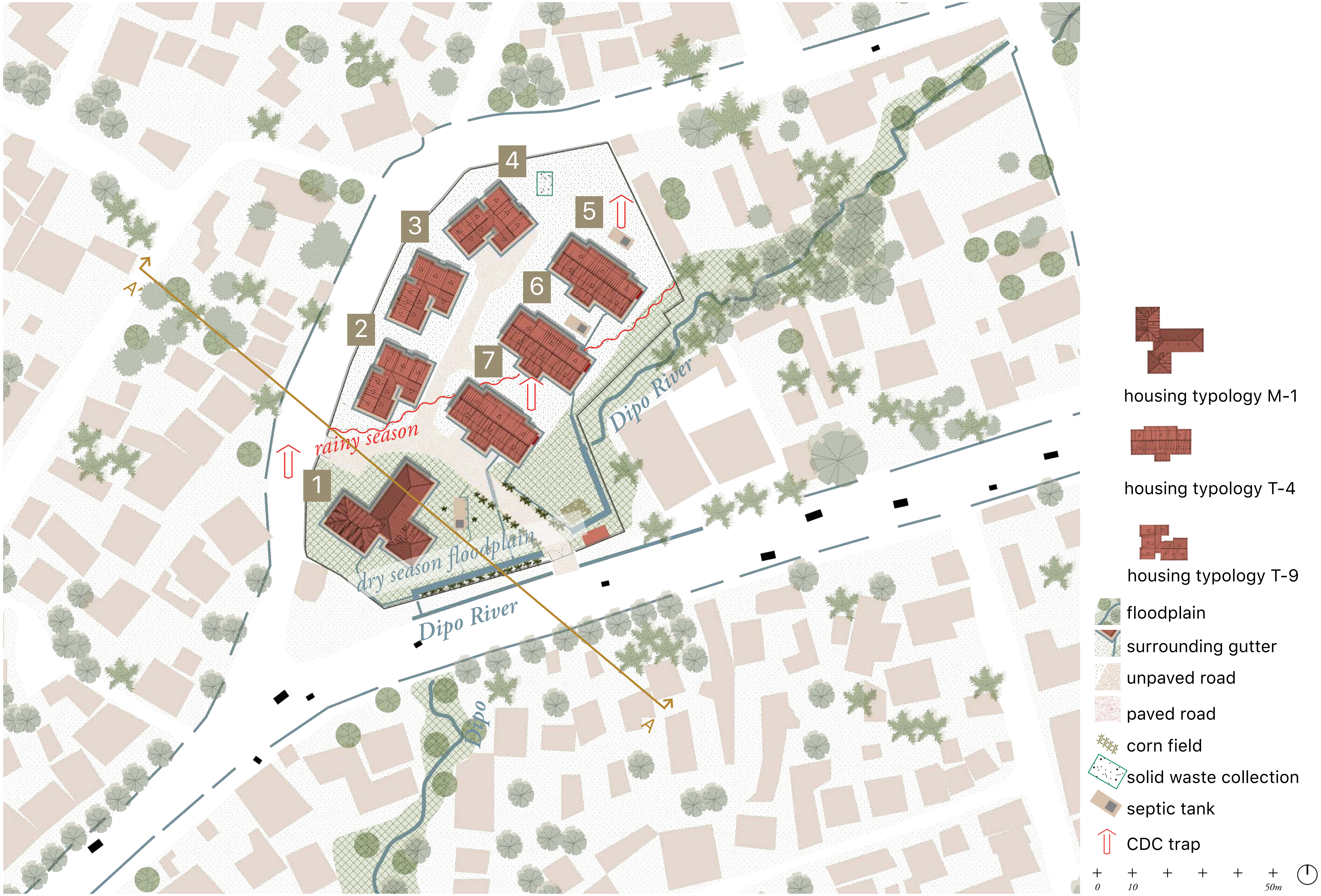

Condominium D

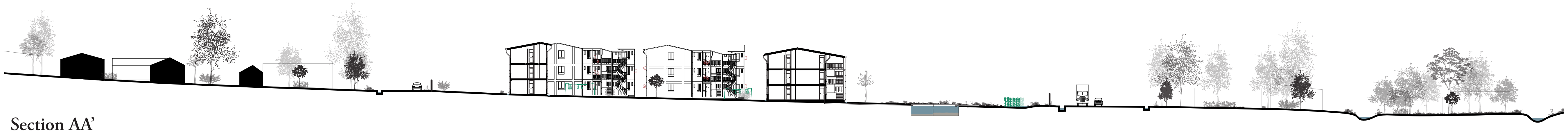

Section AA'

Correspondence:
Stefanie Dens
stefanie.dens@uantwerpen.be

This article was submitted to
Public Health Policy,
a section of the journal
Frontiers in Public Health

Drawings as tools to (re)imagine space in interdisciplinary global health research

2022 Stefanie Dens, Claudia Nieto-Sanchez, Mario De Los Santos, Thomas Hawer, Asgedom Haile, Karla Solari, Jesus Cisneros, Victor Vega, Kalkidan Solomon, Adamu Addissie, Delenasaw Yewhalaw,Larissa Otero, Koen Peeters Grietens, Kristien Verdonck and Maarten Van Acker

FIGURE 8
Jimma, M-scale.

Condominium D

field explorations

- spatial modifications
- social activities
- water network

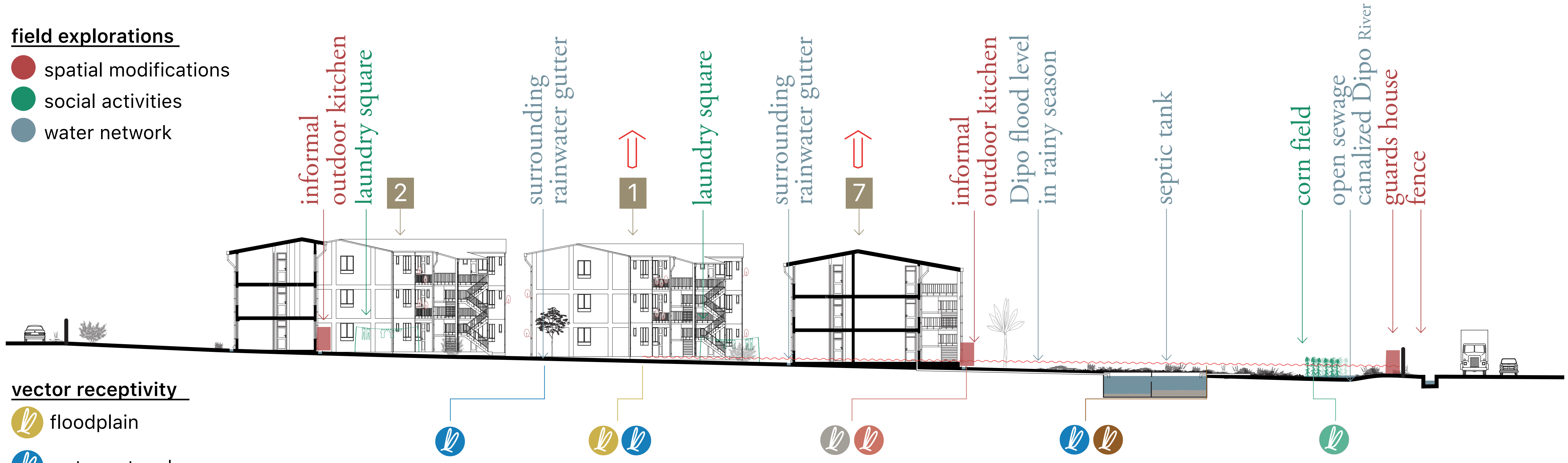

vector receptivity

- floodplain
- water network
- vegetation
- plural lifestyles
- dusk & dawn
- wasteflows

Stagnant water

Debris poored in the surrounding rainwater gutters block the flow of (rain)water

Permanent flooding

The canalised Dipo River frequently floods the lowest areas of this condominium. The ground floor housing units of block 1 flood and are left uninhabited during rainy season.

Outdoor kitchen

The extended kitchen, informally constructed to cook a tranditional meal called 'enjara' is built outdoors over the surrounding rainwater gutter

Septic tank

The septic tanks are often blocked, and flood with rising ground waters or high intensity rains, causing black and grey water to backwash and flow freely on site.

Zea mays (corn)

Corn (Zea mays), plays an important role in mosquito prevalence. Gravid females are attracted to the scent of maize fields and lay their eggs there, and hatched larvae feed on maize pollen.

Zoom of section AA'

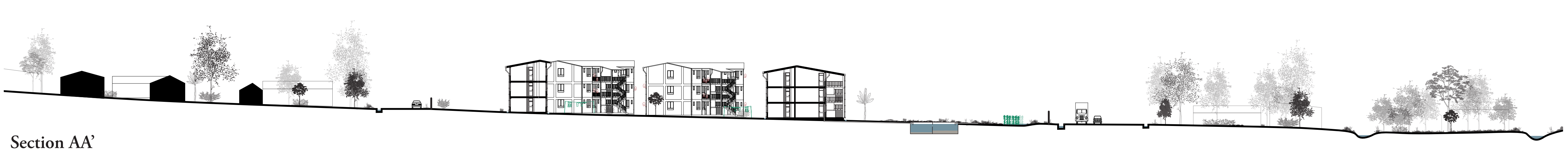

Section AA'

Correspondence:
Stefanie Dens
stefanie.dens@uantwerpen.be

This article was submitted to
Public Health Policy,
a section of the journal
Frontiers in Public Health
